# Therapeutic Strategies in HCC: Radiation Modalities

**DOI:** 10.1155/2016/1295329

**Published:** 2016-08-03

**Authors:** R. Gallicchio, A. Nardelli, P. Mainenti, A. Nappi, D. Capacchione, V. Simeon, C. Sirignano, F. Abbruzzi, F. Barbato, M. Landriscina, G. Storto

**Affiliations:** ^1^Nuclear Medicine Department, Istituto di Ricovero e Cura a Carattere Scientifico (IRCCS), Centro di Riferimento Oncologico di Basilicata (CROB), Via P. Pio 1, 85028 Rionero in Vulture, Italy; ^2^Istituto di Biostrutture e Bioimmagini, Consiglio Nazionale delle Ricerche, 80145 Napoli, Italy; ^3^Oncology Unit, IRCCS Giovanni Paolo II, 70124 Bari, Italy; ^4^CMO Oplonti Medical Centre, 80058 Torre Annunziata, Italy; ^5^Oncology Unit, Università degli Studi di Foggia, 71122 Foggia, Italy

## Abstract

Patients with hepatocellular carcinoma (HCC) comply with an advanced disease and are not eligible for radical therapy. In this distressed scenario new treatment options hold great promise; among them transarterial chemoembolization (TACE) and transarterial metabolic radiotherapy (TAMR) have shown efficacy in terms of both tumor shrinking and survival. External radiation therapy (RTx) by using novel three-dimensional conformal radiotherapy has also been used for HCC patients with encouraging results while its role had been limited in the past for the low tolerance of surrounding healthy liver. The rationale of TAMR derives from the idea of delivering exceptional radiation dose locally to the tumor, with cell killing intent, while preserving normal liver from undue exposition and minimizing systemic irradiation. Since the therapeutic efficacy of TACE is being continuously disputed, the TAMR with ^131^I Lipiodol or ^90^Y microspheres has gained consideration providing adequate therapeutic responses regardless of few toxicities. The implementation of novel radioisotopes and technological innovations in the field of RTx constitutes an intriguing field of research with important translational aspects. Moreover, the combination of different therapeutic approaches including chemotherapy offers captivating perspectives. We present the role of the radiation-based therapies in hepatocellular carcinoma patients who are not entitled for radical treatment.

## 1. Introduction

Hepatocellular carcinoma (HCC) is among the most common tumors worldwide and although most cases still occur in developing countries in South-East Asia and Africa, its incidence is also increasing in developed industrialized states [1–3]. Almost 700,000 new cases are diagnosed yearly throughout the world and unfortunately the prognosis remains poor since more than 500,000 deaths are ascribed to HCC each year [2]. Where the incidence of HCC is rising this datum seems to be related to the better diagnostics and healthcare of patients complying with cirrhosis and the significant increase in chronic hepatitis C. Noteworthy, a significantly higher mortality related to HCC hepatitis- and cirrhosis-based disease is expected in women in next years [4–6]. Contamination of foodstuffs by aflatoxins in some areas cannot also be excluded [7–9]. In this context, only a limited number of cases are eligible for decisive treatments, such as resection, transplantation, ethanol injection, or radiofrequency ablation, since these curative approaches are also indeterminate. On the other hand, a plethora of palliative/alternative treatments has been recently proposed for patients who are not entitled for radical therapy or those who exhibit inoperable locoregional disease without distant metastases. Since in this setting systemic chemotherapy and the conventional external beam radiation therapy (RTx) have been demonstrated ineffective in terms of both survival and safety, transarterial chemoembolization with Lipiodol (an oily medium; TACE) and the transarterial metabolic radiation therapy (TAMR) have proven useful [10, 11]. However, the therapeutic efficacy of chemoembolization is being continuously disputed, both in retrospective and randomized studies [12, 13]. Also, this approach has been demonstrated to provide a similar overall survival when compared to transarterial ethanol ablation, but with a worse complete tumor response, a shorter time to progression, and a shorter progression free survival. Moreover, TACE is associated or not with eluting drugs and seems to not offer significant advantages in comparison with a wait and watch approach [14–17]. Lastly, only few data support the value of the concurrent intra-arterial chemotherapy with external beam radiotherapy (CCRT) when compared to TACE alone [18]. This distressed scenario has endorsed the use of metabolic radiotherapy via hepatic artery administration by labeling Lipiodol with several radioisotopes [19–23] and using Yttrium-90 microspheres or radiolabeled antibodies as valuable tools to attain adequate therapeutic response [24, 25]. The rationale of TAMR derives from the idea of delivering exceptional radiation dose locally to the tumor, with cell killing intent, while preserving healthy liver and the rest of organs from undue exposition. Moreover, the dual vascularization of the liver represents an added value when treating hypervascularized neoplasms such as HCC whose hematic support is mainly provided from the hepatic artery. Among others, Lipiodol has been labeled with ^131^I since 1964 [26] and, more recently, implemented in the management of HCC as well with ^188^Re [21, 27]. Both radioisotopes have been used in humans showing variable response rates. Recently, ^90^Y microsphere therapy has been implemented in preliminary randomized studies with encouraging results in terms of target lesion response as well as overall survival, despite some toxicities [28, 29]. Recent technological advances in the field of RTx, such as the three-dimensional conformal definition of the target volume, make it suitable, alone or in combination, for patients complying with inoperable HCC [30].

## 2. Basic Pathophysiology and Rationale of Radiation Modalities

**Figure 1:**
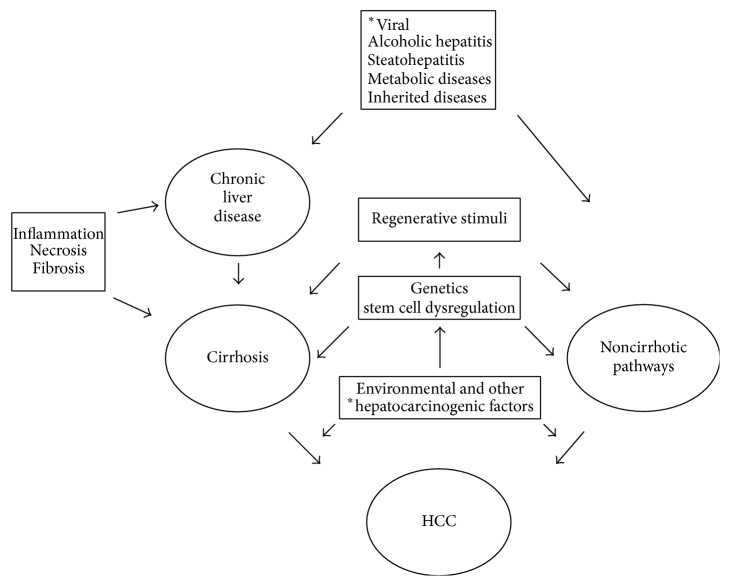
Basic pathophysiology of hepatocellular carcinoma (HCC). Viral, infections causing chronic hepatitis (type B, type C, type D, and others). Metabolic diseases, that is, alpha-1-antitrypsin deficiency. Inherited diseases, that is, Wilson disease and hemochromatosis. Environmental, that is, aflatoxins. *∗*: related to.

The pathophysiology of HCC is a multifactorial event. Inflammation, necrosis, fibrosis, and continuing regeneration characterize both chronic liver disease and cirrhotic liver and may contribute to HCC development in a crescendo. However, dysplastic and regenerative nodules found in cirrhotic livers do not necessarily sustain a progression to HCC, depending on the kinds of proliferating cells (small-cell dysplastic nodules increase risk more than large-cell nodules). Although hepatitis B virus (HBV) was initially supposed to be the primum movens, subsequent studies failed to identify HBV infection as the main independent risk factor [31]. In most cases HCC develops in patients with underlying cirrhotic liver disease of various etiologies, including patients whose HBV infection markers are negative [32]. On the other hand, in patients with hepatitis C virus, HCC frequently occurs in the setting of cirrhosis. The transformation process also encompasses a multitude of pathways that can be modified by external and environmental factors. Finally, primary genetic changes may be the cause of delayed apoptosis and increased cellular proliferation but also result from hepatocarcinogenetic factors (Figure 1). From a radiotherapeutic point of view, radiation delivery to liver lesions is significantly limited by the tolerance of surrounding normal liver parenchyma. Although the underlying cause of hepatic radiation toxicity is a venocclusive disease, the ultimate damage and the risk of developing complication depend on how functional subunits (acini in liver) of organs are structured. Liver is a typical example of a radiobiologically parallel architecture model. As a result, the increased risk of complication depends on dose distribution throughout the whole organ and the exceeding patients' functional reserve rather than the maximum dose to a limited area. Unlike external radiation therapy, internal radiotherapy may convey radioisotopes (and radiation) either percutaneously or through transarterial approach acting as radioembolization. In particular biodistribution and metabolic studies in TAMR have shown that radiopharmaceuticals are fixed in the liver, tumors, and lungs (arteriopulmonary hepatic shunts) the unbound amount (quote; quantity) is eliminated mainly through the urinary tract (30–50% of injected activity) [33]. The rationale of TAMR derives from the idea of delivering exceptional radiation dose locally to the tumor, with cell killing intent, while preserving healthy liver and the rest of organs from undue exposition. Moreover, the dual vascularization of the liver represents an added value when treating hypervascularized neoplasms such as HCC whose hematic support is mainly provided from the hepatic artery. In fact, the hepatic artery supplies 30% of the blood flow to the normal liver parenchyma but greater than 90% to hepatic tumors.

## 3. The Role of Ionizing Radiations in the Management of HCC

Transarterial metabolic radiotherapy plays a crucial role in the management of patients with primary and metastatic liver malignancies. In particular, the efficacy of these locoregional radiation-based treatments has been demonstrated in HCC, which is recognized to be a systemic chemo-resistant cancer, making treatment of these tumors widely approved in the field of interventional radiooncology. This kind of internal radiotherapy is based on the locoregional delivery of radioisotopes either percutaneously or throughout transarterial approach. The ^131^I labeled Lipiodol was the first implemented agent and attempts to use it in humans were performed since 1986 [34]. Lipiodol is an oily medium based substance which has been shown to be selectively retained in tumor when administered intra-arterially and had been originally used as a contrast agent for diagnosing HCC [35]. Even if the mechanism of retention of Lipiodol within the HCC environment is not completely understood, it seems related to the different arteriolar density between the hypervascular HCC and the normal liver parenchyma, to the accumulation into peritumoral sinusoids, to the penetration and slower wash-out from HCC cells [36]. Coupling Lipiodol with the ^131^I radiohalogen has yielded a suitable radiopharmaceutical for treating HCC with emission of beta particles (606 keV), despite some drawbacks (see radioprotection) due to the gamma-ray emission (364 keV; 82% abundance) and the long half-life (8.05 days). However, the gamma-ray emission allows dosimetric estimates. From the beginning this agent has shown a favourable tumor/nontumor liver ratio and several studies have been performed to demonstrate its biodistribution in nontarget organs as well. Reported values of tumor/nontumor ratios are between 4.3 and 20 allowing the delivery of a significant amount of irradiation to the tumor. Moreover, the retention of ^131^I Lipiodol shows an inverse correlation with the tumor size and its effective half-life was reported to be approximately 4.6 days [37, 38]. Alternatively, both ^188^Re Lipiodol (beta emission 2.1 MeV, half-life of 16.9 hours, and presence of gamma emission 155 keV energy, suitable for dosimetry) and ^90^Y Lipiodol (beta emission 2.2 MeV, half-life of 2.67 days, and tissue range penetration of 2.5 mm) have also been used because of the favourable characteristics of these radioelements [39, 40], but some studies have been limited to animals or failed to demonstrate adequate safety and efficacy (Table 1) [41, 42].

Recently, microspheres labeled with ^90^Y have been used in several clinical studies with encouraging rates of response. The development of the microspheres composed of resin or glass containing this radioelement in a stable form avoided the toxicity problems encountered at the beginning due to the systematic release of the radionuclide [43–45]. The favourable radiochemical and therapeutic characteristics of ^90^Y, especially when using high activity levels [46], have resulted in the increasing use of this agent similar to use of ^90^Y for radioimmunotherapy of lymphoma [47].

Although a standardized RTx does not exist for liver, technological advances in the field of treatment planning have recently provided the means for delivering appropriate killing doses to a defined liver lesion, achieving satisfactory therapeutic ratios [48]. In this way, complications became acceptable. These novel irradiation techniques include the three-dimensional conformal radiotherapy for the accurate definition of the HCC target volume as well as of surrounding organs [49]. Treatment plans use multiple fields to irradiate the tumor and spare normal tissues. The above-mentioned RTx has been used for cirrhotic HCC patients not eligible for radical therapies, with encouraging results [50, 51]. Moreover, some studies have indicated that stereotactic body radiation therapy is a safe and effective modality treatment for HCC [52–57]. This new therapeutic approach has gained an outstanding importance since it can be a bridge modality in patients who are candidate for transplantation [58, 59].

## 4. Indications, Contraindication, and Special Precautions

According to the pharmacokinetics of radio-coupled Lipiodol and microspheres, they can be properly indicated in patients having HCC with portal vein thrombosis [60, 61]. Such types of therapies can be also be employed as adjuvant treatment near the surgical resection, reducing the risk of recurrence [62]. However, this agent seems to be more effective when it is used in a neoadjuvant approach, since surgery can provoke the release of growth factors while neighbouring synchronous metastases could be comprised in the therapeutic plan [63]. Additional indications are the palliative treatment of not operable HCC, though it should be preferentially implemented in patients without distant metastases.

In general, the treatment should be reserved to HCC nodules less than 6–8 cm in diameter. It could be implemented as curative therapy of small-size HCC lesions which are inaccessible or as neoadjuvant treatment before hepatic transplantation in order to reduce the risk of recurrence on the graft. Finally, the two other indications can be evoked for TAMR and include the reduction of tumor burden in case of HCC that are too large to be operated and as antalgic treatment for patients who suffer from hyperalgesic HCC not more responsive to conventional analgesia. Concerning the external radiation approach, the patients could be eligible in case of HCC (either primary or recurrent) when surgery or percutaneous ablative therapies are unfeasible and difficult or patients demonstrate a refusal. In addition, patients presenting the longest tumor diameter of ≤5.0 cm and those exhibiting Child-Turcotte-Pugh Class A or B (no severely compromised liver function) can be considered. In particular, external beam radiotherapy can be safely considered for patients with 1–3 lesions and sufficient uninvolved liver parenchyma [64–67].

Contraindications of TAMR include the presence of distant metastases and patients complying with evolved cirrhosis or locally very advanced HCC and/or the presence of comorbidities as severe respiratory or renal insufficiency as well as bone marrow suppression (to be evaluated also in case of external radiation therapy). Moreover, patients with a documented pathology of the aorta or femoral arteries with iodine allergy and pregnancy status cannot be considered for this therapy. Finally, other contraindications embrace patients who had previous RTx to the liver or those treated with capecitabine within the two previous months. Patients who are scheduled to receive capecitabine at any time following treatment with ^90^Y resin microspheres and those having greater than 20% lung shunting of the hepatic artery blood flow cannot be enrolled for TAMR. The lung represents a critical organ in the implementation of transarterial metabolic radiotherapy since pulmonary uptake rises in reason of a secondary release of the Lipiodol from the liver or in presence of arteriopulmonary hepatic shunt. In the case of ^90^Y microspheres, the excessive irradiation may result in radiation hepatitis. An inadvertent delivery of microspheres to the gallbladder or to the gastrointestinal tract or pancreas can effect, respectively, a cholecystitis and pancreatitis. Also, a high level of implanted radiation and/or excessive shunting to the lung may lead to radiation pneumonitis. In the course of radiation therapy, asthenia, anorexia, and transient hepatalgies rise with temperature and a brief alteration of hepatic tests and leucopoenia should be considered, whereas prolonged fever, severe hepatic insufficiency, and gastroenteric haemorrhages are serious but rare side effects (as shown below).

Contraindications from commonly used ionizing radiation-based therapies (internal and external) for HCC are the following:

- Distant metastases (others than liver).
- Evolved cirrhosis or locally very advanced HCC.
- Severe respiratory or renal insufficiency.
- Bone marrow suppression.
- Documented pathology of the aorta or femoral arteries (internal).
- Iodine allergy (^131^I Lipiodol).
- Pregnancy status.
- Previous external beam radiation therapy to the liver.
- Previous treatment with capecitabine within the two previous months or treatment with capecitabine at any time following treatment (^90^Y microspheres).
- Greater than 20% lung shunting of the hepatic artery blood flow (internal).

## 5. Administration and Treatment Sequence

The two ways of administration that can be performed include selective injection into a hepatic artery via gastroduodenal artery or hyper-selective injection at the right- or left-hand side of the hepatic artery to the depth of the subsegmental arteries [68, 69]. The last vascular access, once multiple foci have been excluded, allows an increase of delivered radiation dose to the tumor sparing normal liver and lungs. According to different studies, the administered activity generally ranges from 0.74 to 4.40 GBq for ^131^I Lipiodol and from 2.0 to 3.0 GBq for ^90^Y microspheres, respectively [70]. Therapeutic activities have also been settled on the basis of dosimetry and alternatively computed taking into account the dose delivered to the target or to the critical organs [71, 72]. On the other hand, the super-selective approach excludes the prophylactic irradiation of the healthy parenchyma that has been established effectively in reducing relapse or recurrence. From a methodological point of view, this approach requires a slow injection in case of small diameter artery in order to avoid reflux (flow rate to be used: 10–20 mL per hour).

The injection is carried out at the time of hepatic arteriography according to the technique of Seldinger [73]. The left femoral artery is the preferred access route in order to reduce operators exposure, even if standardized administration protocols do not exist. When ^90^Y labeled microspheres are used, hepatic scintigraphy after the injection into the hepatic artery of macroaggregates of Tc-labeled human albumin is required. The scan allows evaluation and quantification of pulmonary shunts, if any, and of gastrointestinal shunts. It can also be used to estimate the dose to both the target and critical organs as well. Using ^131^I labeled Lipiodol, it is mandatory to hospitalize the patients in a protected room (see radioprotection) and to perform an abdominal scintigraphy searching for tumor retention and pulmonary uptake. Injection can be repeated every two-three months in patients presenting with large tumors and supplementary injections could be implemented when an objective response has been demonstrated. In general, the effectiveness of retreatment, if any, depends mainly on the amount of Lipiodol or microspheres retained by the tumor. Lastly, succeeding treatments can also be implemented in case of multiple foci with the intent to cure patients who initially presented with a weak retention after the hyperactive areas are destroyed. Regarding external beam radiation therapy, it should be considered that HCC is generally not a radio-resistant tumor but is located in an extremely radiosensitive organ. Accordingly, the dose delivered should not exceed 30 Gy on the whole liver as this is the threshold for radiation-induced liver disease. This dose level is less than standard tumor-killing doses for most of the other radiosensitive neoplasms. Advances in the field of radiotherapy planning have given the opportunity of dose escalation.

## 6. Dosimetry and Safety on Workplace

The rationale of radiotherapy includes the fact that the dose to be delivered for a therapeutic effect should be computed taking into account the dose delivered to critical organs. In case of ^131^I Lipiodol the accepted killing dose for HCC is 120 Gy, being normal liver and lungs the critical organs.

For external beam radiotherapy it depends on fractionation and the total irradiation of liver and lungs should not exceed 30 Gy and 25 Gy, respectively [74], whereas the dose to critical organs is not exactly computable in case of metabolic radiotherapy. However, when ^90^Y labeled microspheres are used the mean adsorbed dose in the liver is 120 Gy and more than 30 Gy in lungs. Apart from the radiometric issues related to the use of radioiodine (see body and hands exposure), it should be pointed out that the use of ^90^Y may cause consistent exposure to the fingers of the personnel involved in the preparation and administration of the radiopharmaceuticals.

## 7. Clinical Trials

Considering ^131^I Lipiodol, a partial or incomplete response (reduction of tumor size and/or of hematic markers) was found in almost 71% of patients [75, 76]. These results were confirmed also after correcting for the number of patients per study [77]. A complete response has been also described depending on type and HCC size. Multinodular forms showed a lower rate of response as well as those infiltrating. It has been demonstrated that the response rate is inversely correlated to the size of HCC, being 80% for nodules of less than 5 cm diameter [78] up to 22% for those of 10 cm. However, the sole dimensional assessment of therapeutic response is not accurate since metabolic change should be taken into account. Accordingly, recent studies have shown the usefulness of positron emission tomography/computed tomography (PET/CT) for assessing the response to locoregional treatment of HCC [79] as demonstrated for other tumor types [80] and considering the potentialities of PET/CT methodology [81]. Moreover, other studies reported responses in 17%–92% of patients after ^131^I Lipiodol intra-arterial administration [77, 82]. Lintia-Gaultier et al. compared fifty patients with advanced HCC receiving intra-arterial injection of ^131^I Lipiodol with 36 untreated patients. The ^131^I Lipiodol was associated with a survival benefit (32 wk versus 8 wk for the untreated group) and the 1-year survival rate was 32% versus 8% for the untreated group [19]. The transarterial metabolic radiotherapy has also been used for reducing the pain related to HCC whereas, in general and according to different trials, survival rates of 6 months are reported between 33 and 66% [83]. ^90^Y microspheres can be used for treatment of large tumors of patients eligible for transplantation, in patients with portal vein thrombosis and with palliative intent [84]. The radioembolization of unresectable HCC with ^90^Y microspheres is associated with acceptable toxicity and a favourable median survival time [85]. The intra-arterial administration of ^90^Y microspheres has shown safety and effectiveness in both intermediate-stage and advanced-stage HCC [86]. This trend has been recently reported in retrospective and prospective studies [87–90]. The efficacy of external radiation therapy has been demonstrated in patients with small-size HCC noneligible for curative therapies [91]. Data from Mornex et al. (phase II study) confirmed the efficacy of radiation therapy in cirrhotic patients with one nodule ≤5 cm or two nodules ≤3 cm. Tumor response was observed for 92% overall with 80% of complete response. After a mean follow-up of 29 ± 9 months, the recurrence rate was 22% and 41% for lesions inside and outside the irradiated volume, respectively [92].

Multicenter studies sponsored by the International Atomic Energy Agency (IAEA) showed that transarterial ^188^Re Lipiodol is a safe and cost-effective method to treat primary hepatocellular carcinoma. Immediate and late side effects were minimal. The objective response rate was 25% and 1- and 2-year survivals were 46% and 23%, respectively [39, 40].

## 8. Still Open Questions and Combination of Therapies for HCC

The efficacy of ^131^I Lipiodol has been evaluated versus chemoembolization. Patients complying with inoperable and nontransplantable HCC randomly underwent ^131^I Lipiodol or chemoembolization evaluating tumor size, alpha-fetoprotein levels, and survival rate. There was no significant difference in survival rate at 6 months and at four years between the two treatments as well as in terms of complete response. Both therapeutic methods showed similar effectiveness, although the ^131^I Lipiodol group exhibited better tolerance to treatment [93]. ^131^I Lipiodol was also shown to improve significantly the survival when compared to supportive medical therapy [63]. It seems clear that TAMR with ^131^I Lipiodol is at least as effective as the TACE, showing a better safety profile. This treatment has been also used with adjuvant intent and patients compared to a control group. The survival rate at 3 years in the treatment group and controls was 86.4% and 43.6%, respectively [62]. ^131^I Lipiodol has also been combined with low-dose of cisplatin and patients who received both treatments showed a response rate of 90%, as compared to those who received ^131^I Lipiodol alone [94]. This combined approach is important and a synergy between radiotherapy and chemotherapy can be envisaged. This approach was already tested using ^90^Y microspheres in patients with hepatic metastases [95]. Actually, there are no published studies endorsing the combination of ^131^I Lipiodol with external radiotherapy, but indeed it represents an intriguing area of research similar to that combining hyperthermia with transarterial metabolic radiotherapy. Recently, the efficacy of ^90^Y microspheres has been compared with transarterial chemoembolization for treating HCC [96]. No significant differences were found in progression free survival, overall survival, and time to progression. The lower rate of tumor progression in radio-treated group was nullified by a greater incidence of liver failure [96]. Both these modalities can be alternatively used in case of unresectable HCC, as first-line treatment [97]. Concerning the combination of ^90^Y microspheres with other treatment modalities, a plethora of studies has recently shown that radioembolization followed by an inhibitor of tyrosine protein kinases (sorafenib) appears to be as well-tolerated as the inhibitor alone [98–100]. Conformal radiotherapy combined with hepatic arterial floxuridine has improved the survival of patients with intrahepatic cancer ineligible for surgical resection or ablation as reported by Ben-Josef et al. [98]. Moreover, the total radiation dose was the only significant factor for survival. As for other cancer types and other aggregate therapies, a cumulative tumor-killing effect can be postulated when using a combination of RTx and inhibitor of tyrosine protein kinases [101, 102]. This captivating area of research (Table 2) demands further preclinical and clinical trials in order to offer new therapeutic options for HCC (Table 3).

## 9. Economics 

The cost of the commercially available radiopharmaceuticals ^131^I Lipiodol and ^90^Y microsphere is prohibitively high for developing countries where the HCC represents an impending social problem. The actual economic context requires that a treatment should be cost-effective, easy to transport, and uncomplicated to administer and should not create radiation protection issues. All these topics contribute to the global cost of transarterial metabolic radiotherapy for hepatocellular carcinoma, each of them with a different but definite weight. The current price of ^188^Re generator can be considered convenient for developed countries, but it will constrain wide clinical application of ^188^Re in developing countries. In general, the accurate assessment of these costs is complicated. However, it should be pointed out that radiation-based therapy for HCC determines less side effects as compared to other modalities reducing the costs for supportive therapies and warranting adequate quality of life.

## 10. Conclusion

The role of transarterial metabolic radiotherapy for hepatocellular carcinoma has gained full consideration over the years. ^131^I Lipiodol and ^90^Y microspheres have proven effectiveness in the treatment of inoperable HCC also with portal vein thrombosis. This therapy can also be implemented with either neoadjuvant or adjuvant intent, reducing the risk of recurrence. It has been recognized as effective as chemoembolization. The transarterial metabolic radiotherapy is more effective than palliative supportive therapies and can be used for dealing with pain in the event of hyperalgesic HCC. Contraindications are clearly defined and side effects are controllable and predictable, supporting this treatment as being well-tolerated. Radiation protection and manipulation issues are manageable. Transarterial metabolic radiotherapy alone or in combination with other local or systemic therapies offers encouraging results on tumor local control and survival. The literature also supports the efficacy and safety of external radiation therapy for HCC that has until now been considered a radio-resistant tumor.

## Figures and Tables

**Table 1:** Examples of radioisotopes used for HCC transarterial metabolic radiotherapy.

Radioelements	Half-life (days)	Maximum beta energy (MeV)	Maximum range in tissues (mm)	Gamma energy (KeV)
Iodine-131	8.05	6.06	2	364
Rhenium-186	3.7	1.7	5	137
Rhenium-188	0.7	2.1	10	155
Yttrium-90	2.67	2.2	12	None
Holmium-166	1.1	1.85	8.7	80.6

**Table 2:** Therapeutic strategies for nonoperable HCC.

Treatment	Indications	Advantages	Disadvantages	Efficacy
TACE	(i) Large or multifocal HCC not eligible for radical therapy (ii) Nonoperable locoregional disease without distant metastases	(i) Safety (ii) Bridge modality in patients awaiting OLT	(i) Poor complete tumor response (ii) Short time to progression (iii) Short progression free survival (iv) PES (v) Ischemic damage to normal liver	(i) Tumor shrinking (ii) Survival (moderate)

TAMR	(i) Curative therapy of small-size HCC (ii) HCC with portal vein thrombosis (iii) Adjuvant treatment near surgery (iv) Neoadjuvant approach (v) Palliation of nonoperable HCC (vi) Antalgic treatment for hyperalgesic HCC	(i) Cell killing intent (ii) Healthy liver preservation (iii) Bridge modality in patients awaiting OLT	(i) Radiation pneumonitis/hepatitis/cholecystitis/pancreatitis (ii) Asthenia, anorexia, transient hepatalgies, brief alterations of hepatic tests, and leucopoenia (iii) Workplace safety	(i) Tumor shrinking (ii) Survival benefit (iii) Long term efficacy

RTx^*∗*^	(i) Nonoperable HCC (ii) Unfeasible/failed percutaneous therapies (iii) Longest tumor diameter ≤5.0 cm (iv) Child-Turcotte-Pugh Class A or B (v) Patients with 1–3 lesions and sufficient uninvolved liver	(i) Bridge modality in patients awaiting OLT (ii) Dose escalation (iii) Accurate definition of the target volume	(i) Surrounding healthy liver damage	(i) Significant tumor response in small-size HCC

TACE: transarterial chemoembolization. OLT: orthotopic liver transplantation. PES: postembolization syndrome. TAMR: transarterial metabolic radiotherapy. RTx: external radiotherapy. **∗**: including 3D conformal radiation techniques and stereotactic body radiotherapy.

**Table 3:** Summary of recent clinical trials considering radiation-based therapies (internal and external) for HCC.

Reference	Year	Therapy	Findings
[75]	1988	^131^I Lipiodol	50% tumor size reduction, response rate 60%
[77]	1991	^131^I Lipiodol	Response rate 88.9%–25% according to tumor size
[83]	1992	^131^I Lipiodol	Decrease of pain in 33%–66%
[93]	1997	^131^I Lipiodol versus TACE	^131^I Lipiodol and TACE equally effective
[95]	2001	^90^Y microspheres plus CHx versus CHx alone	Tumor response 44% combined therapy versus 17.6% CHx alone
[94]	2002	^131^I Lipiodol plus cisplatin	Response rate 90% combined therapy versus 40% ^131^I Lipiodol alone

[91]	2005	RTx	Tumor response 66.1%
[98]	2005	RTx plus floxuridine	Improved survival in patients with unresectable intrahepatic malignancies
[82]	2005	^131^I Lipiodol	Response rate 17%–92%
[87]	2006	^90^Y microspheres	Disease control rate 100% and response rate 23.8%
[92]	2006	RTx	Response rate 92% in patients with small-size HCC
[39]	2008	^188^Re Lipiodol	objective response rate 25% 2-year survival 23%

[85]	2012	^90^Y microspheres	Favourable median survival time

[19]	2013	^131^I Lipiodol	Survival benefit; 32% of treated versus 8% of untreated pts

[99]	2014	^90^Y microspheres plus sorafenib	Potential efficacy and manageable toxicity
[86]	2015	^90^Y microspheres	Safe and effective in both intermediate- and advanced stage
[96]	2015	^90^Y microspheres versus TACE	No significant differences in PFS, OS, and TTP
[100]	2015	^90^Y microspheres plus sorafenib	^90^Y microspheres plus sorafenib well-tolerated as sorafenib alone
[88]	2016	^90^Y microspheres versus sorafenib	^90^Y microspheres more effective than sorafenib in patients with PVT

Radionuclides administration by transarterial approach. TACE: transarterial chemoembolization. CHx: chemotherapy. PFS: progression free survival. OS: overall survival. TTP: time to progression. PVT: portal vein thrombosis. RTx: external radiotherapy including conformal radiation techniques.

## Additional Points

Core tips: treatment of hepatocellular carcinoma is challenging since it is frequently associated with a causal liver morbidity. The role of radiation-based locoregional therapy has progressed regardless of few side effects. Radiopharmaceutical and technical advances hold great promise for patients who are not eligible for radical treatments. The combination of different therapeutic approaches constitutes an interesting option as well.
